# Early-life short-term environmental enrichment counteracts the effects of stress on anxiety-like behavior, brain-derived neurotrophic factor and nuclear translocation of glucocorticoid receptors in the basolateral amygdala

**DOI:** 10.1038/s41598-020-70875-5

**Published:** 2020-08-20

**Authors:** Akshaya Hegde, Shruti Suresh, Rupshi Mitra

**Affiliations:** School of Biological Sciences, Nanyang Technological University, Singapore, 637551 Singapore

**Keywords:** Emotion, Stress and resilience

## Abstract

Early life is a decisive stage for the development of physiological and psychological characteristics of an individual. Any stress or disruption of healthy development at this stage has serious long-lasting consequences for the remaining life. Unfortunately, early life stress is a common occurrence in humans and other animals. In this context, we investigated if the provision of environmental enrichment during the pre-weaning phase of rat pups and dams could alter the consequences of early-life maternal-separation stress. Pre-weaning enrichment rescued the effects of maternal separation on the excess secretion of adrenal stress hormones and anxiety-like behavior during adulthood. Enrichment also reduced the effect of stress on the spine density of basolateral amygdala neurons, a brain region critical for stress-induced facilitation of emotional behaviors. Pre-weaning enrichment, provided during early-life, blunted the effects of maternal separation stress on decreased intra-nuclear translocation of glucocorticoid receptors within the amygdala neurons when tested later in adulthood. Early-life, pre-weaning environmental enrichment also increased the amount of brain-derived neurotrophic factor within adult basolateral amygdala. Our observations showed that environmental manipulation during early formative years could be utilized to build lifelong resilience to stress. Complex naturalistic housing and sensory enrichment is, thus, an useful buffer against an impoverished and stressful childhood.

## Introduction

Early life is an important period for brain development. Experiences during this period often result in a long-lasting realignment of the stress-response for rest of life. Exposure to stress in early life causes a sustained vulnerability to stress-responsive psychiatric disorders, including anxiety, depression, and post-traumatic stress disorder^1,2^. In order to potentially blunt the harmful effects of stress, it is crucial to understand the mechanistic basis of the relationship between early-life stress and later development of emotional disorders^3^.

Maternal separation is a particularly significant and established model of early life stress^4^. Interestingly, the emergence of aversive stress response in pups is moderated by maternal presence through a reduction in adrenal glucocorticoid release and through the reduction in activity of the amygdala within brain^5^. Thus, maternal presence is an important regulator for stress response in rodent offspring; and maternal absence or separation results in long-lasting stress hyper-responsivity.

A perceived threat or an adverse environment leads to the adrenal release of glucocorticoids through the hypothalamic–pituitary–adrenal axis (HPA axis)^6,7^. Regulation of the HPA axis plays a crucial role in restoring homeostasis after exposure to acute stressors^8–10^. HPA axis remains subdued during early life of rat pups between post-natal days 3 through 14, a phase known as stress hypo-responsive period. Early life stress disrupts the HPA quiescence during this period through increasing glucocorticoid secretion, leading to ontogenic disruption of stress-related behaviors and brain regions. The emergence of adult-like fear response after the stress hypo-responsive period is mediated by greater ability of the adrenals to secrete glucocorticoids and the action of adrenal glucocorticoids within the basolateral amygdala^11^. This suggests that synchrony between glucocorticoids and amygdala is critical in healthy normative development of the stress response.

Early life is also a suitable window to institute stress resilience since it is the phase when the environment can create long-term changes in brain development. Environmental enrichment, is known to exert beneficial effects on stress physiology, synaptic plasticity, molecular changes, and behavioral manifestation of stress^12–15^. For example, post-weaning environmental enrichment rescues the effects of early life stress on anxiety-like behavior and HPA axis^13^. Effects of environmental enrichment during pre-weaning stages, a stage when maternal presence is an expectant event, remains underexplored. This opens a possibility that short-term environmental enrichment provided to the mother–pup dyad early in life may counter the damaging effects of maternal separation on emotional reactivity and commensurate plasticity in the brain during adulthood.

Anxiety is a common maladaptive behavioral outcome of stress. In this context, earlier work demonstrated the central role of structural changes within the basolateral amygdala in stress-induced anxiogenesis^12,13^. Additionally, elevated secretion of adrenal glucocorticoids is a reliable indicator of stress hyper-sensitivity. Glucocorticoids are also known to interact with the basolateral amygdala neurons^16^, specifically through structural plasticity in the dendrites. These interactions are essential for fear and anxiety. The importance of glucocorticoid-mediated signaling is evident through the observation that blocking of glucocorticoid binding to its receptors within the basolateral amygdala reduces stress-induced anxiogenesis^17,18^. Similarly, the provision of glucocorticoids within amygdala enhances fear and anxiety. Apart from glucocorticoids, neurotrophins like brain-derived neurotrophic factor (BDNF) are essential regulators of stress-induced neuronal plasticity. GR and BDNF are known to interact with each other, with BDNF facilitating GR signaling and consequent gene transcriptional response to stress through glucocorticoid response element (GRE)^19–21^.

In this backdrop, we investigated if maternal separation causes coordinated long-term changes in stress response, anxiety-like behavior, basolateral amygdala structural plasticity, regulation of GR activation and regulation of BDNF within the basolateral amygdala. We also investigated the ameliorative effects of a short-term sensory environmental enrichment on all of these parameters.

## Materials and methods

### Animals

Rats of Wistar strain were procured from Charles River, USA. Animals were maintained two per cage in a 12:12 light–dark cycle (lights on at 0700 h). Food and water were available ad libitum. All procedures were approved by the Nanyang Technological University Institutional Animal Care and Use Committee (IACUC). All animal experiments were congruent with and were performed in accordance with the guidelines of the IACUC.

At the start of the experiments, breeding pairs were set by co-housing one male and one female rat. Both mating partners were sexually inexperienced. Male was removed after confirmation of pregnancy at approximately two weeks after the start of the co-housing. Day of birth was designated as PND0 (post-natal day 0). Pups were weaned on PND21.

### Experimental treatments

All experimental treatments were conducted between PND2 and PND21 (post-natal day 2 and 21). During this time, experimental subjects were not weaned from the dams. Pups remained unhandled during the course of the experiment, except during the maternal separation procedures. Experimental animals were weaned at PND21. All behavioral, physiological, and histological measurements were conducted when these weaned animals reached adulthood (PND60 through PND70). Experimenters were blind to the treatment groups during the measurement of endpoints. Only male animals were used for the quantification, although litters remained unmanipulated and consisted of pups of both sexes.

**Figure 1:**
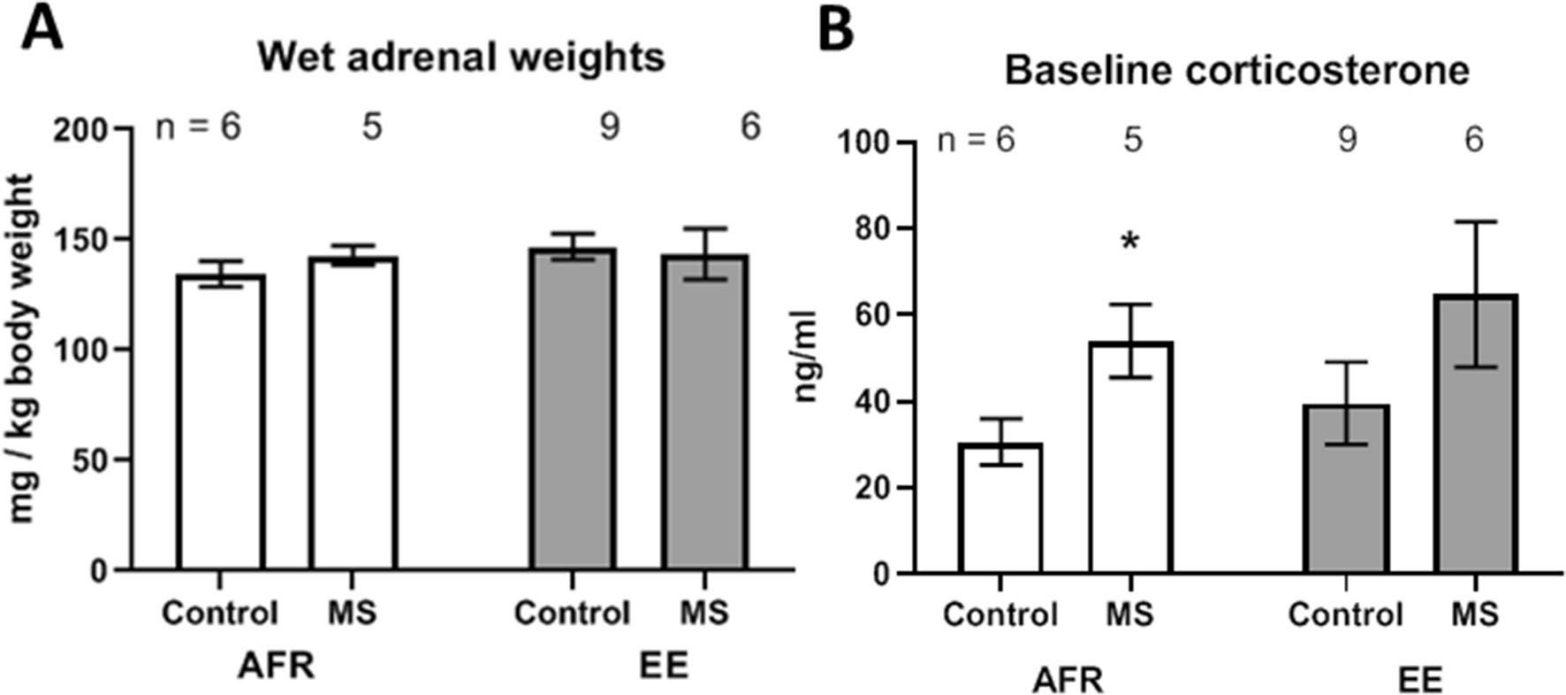
(**A**) Effects of maternal separation (MS) and environmental enrichment (EE) on wet weight of adrenal glands and (**B**) baseline circulating corticosterone. Graphs depict Mean ± SEM. *p < 0.05; ***p < 0.001. Both MS and EE were provided pre-weaning, while endpoints were quantified during adulthood. n refers to number of animals.

Pups assigned to receive maternal separation were separated from the dams once a day for three hours from PND2 through PND14 (post-natal day 2 through 14). The dam was first gently removed and placed in a new cage with fresh bedding. The pups were then removed and placed into a new cage with fresh bedding as a group, brought into a neighboring room, and placed on a heating pad for three hours. Both pups and dams were returned to the home-cage after three hours. Soiled bedding was changed on PND2, PND9, and PND14, by returning pups and mother to a new cage with fresh bedding, which had been previously sprinkled with a handful of soiled bedding and nesting material from the previous cage. Adult animals that received pre-weaning maternal separation were designated as 'MS', and those without maternal separation were designated as 'Control’ in this manuscript (for example, Fig. 1).

Pups assigned to receive environmental enrichment (EE) were placed in enriched housing between PND2 through PND21. One dam and her litter were placed in one enriched housing cage that was much bigger in dimension (72 × 51 × 110 cm^3^ compared to standard of 37 × 22 × 18 cm^3^) and provisioned with a highly sensory environment. A sensorially rich environment was created within the cages by the provision of nesting materials, wooden climbing planks, metal climbing chains, bells, burrowing tubes of various sizes, enrichment toys, and fruit-flavored chow. The EE cages also contained wire meshes on the walls for rats to climb. The location of the objects within EE cages was changed every fourth day to maintain novelty. Water and regular rat chow were provided ad libitum. Apart from the regular food, flavored cereals and sunflower seeds were sprinkled every fourth day. The light and dark cycle was similar to that of the standard cages. This sensory enrichment paradigm did not include the provision of a running wheel. Adult animals that received pre-weaning environmental enrichment are designated as 'EE,' and those without maternal separation are designated as 'AFR' (animal-facility reared) in this manuscript (Fig. 1).

Animals in eleven litters were randomly assigned to one of the four experimental groups: with/without maternal separation (MS or Control), raised in either standard housing (AFR) or enriched housing (EE). After weaning, all animals were housed in individually ventilated standard cages with two animals/cage (37 × 22 × 18 cm^3^).

### Corticosterone measurement

50–200 µl of blood was collected through lateral tail vein incisions for baseline corticosterone measurement at P56^22^. Blood was centrifuged (4,700 rpm, 15 min; Thermo Scientific Sorvall Legend XTR, Germany) and the separated serum was stored at – 80 °C until further analysis. The concentration of serum corticosterone was quantified using an enzyme-linked immunoassay based commercial kit (Enzo Life Sciences, NY, USA). Of note, adrenal and endocrine measures reported here reflect the tone of the stress endocrine response and do not provide information about sensitivity to novel stressors. Adrenal glands were collected bilaterally during sacrifice, and their weight were taken, reported as normalized to body weight at sacrifice.

### Open-field test

An open field test was conducted in a circular Perspex arena (radius = 1 m) between 0900 and 1200 h. Animals were placed at the periphery of the dimly illuminated open field for a trial duration of 300 s. Apparatus was cleaned with 70% ethanol between trials. Time spent in the center of the arena was quantified from recorded videos. The center was defined as a concentric circular region of interest with a radius of 0.33 m. In a subset of animals, total time spent by animals being mobile in the arena was also quantified as an index of locomotion.

### Quantification of dendritic spines

The staining methods used in this study were similar to those previously described^12^. In brief, freshly harvested brains were obtained by sacrifice through decapitation sans anesthesia. Blocks of brain tissue containing BLA (Bregma − 2.04 to − 3.36) were processed for Golgi-Cox stain using a commercial kit (FD Neurotechologies, Columbia, USA). After processing, 100 µm thick coronal sections were mounted on glass slides, counter-stained with cresyl violet (Sigma-Aldrich, Singapore) dehydrated using an ascending series of alcohol and xylene, and coverslipped in non-aqueous medium.

Analysis of spine density was performed using methods similar to those previously described^23^. Spine density on primary and secondary dendrites of spiny principal basolateral amygdala neurons was calculated at 1000× optical magnification using an oil-immersion objective lens (Olympus BX43). Dendrites directly originating from cell soma were classified as primary dendrites, and those arising from primary dendrites were classified as secondary dendrites. Starting from the origin of the branch, and continuing away from cell soma, spines were counted along a 60 µm stretch of primary and 60 µm of secondary dendrite. Density was expressed as number of spines per µm, averaged across primary and secondary dendrites.

### Tissue preparation for immunohistochemistry

Anesthetized rats (gaseous isoflurane, 5%) were sacrificed through transcardial perfusion of phosphate-buffered saline followed by 4% paraformaldehyde dissolved in buffered saline. Harvested brains were equilibrated with 30% sucrose in buffered saline. These brains were cryosectioned in coronal planes at 40 µm thickness and collected in a 24-well plate with anti-freeze media and stored at − 20 °C until further use. Three randomly chosen sections between Bregma − 2.76 to − 2.92 mm were used for immunostaining for glucocorticoid receptors. Another set of three sections were used for immunostaining of brain-derived neurotrophic factor.

### Quantification of intra-nuclear glucocorticoid receptors (GR)

Sections were washed in phosphate-buffered saline (3×) and then incubated with 5% bovine serum albumin for one hour. Primary antibody (rabbit anti-GR; Santa Cruz, USA) was diluted (1:500) in a solution containing 0.1% Triton × 100 and 3% bovine serum albumin in buffered saline. Brain sections were incubated with the primary antibody for 48 h at 4 °C with gentle shaking. Sections were thoroughly washed in the buffer after incubation. Localization of primary antibody was visualized using anti-rabbit antibody raised in goat and conjugated with DyLight-549 (1:1,000, Vector Laboratories, USA; 2 h at room temperature). Nuclear boundaries were subsequently stained after washing unbound antibodies and incubating sections with 4′, 6-diamidino-2-phenylindole (DAPI) for 1 min. Sections were washed and mounted in aqueous medium on superfrost slides and stored at − 20 °C until imaging.

Four randomly selected regions of interest (ROI: BLA, Bregma − 2.04 to − 3.36) for each animal was imaged at 400× magnification using the confocal laser scanning microscope LSM 710 (Carl Zeiss, Singapore). 35–40 confocal slices were collected per region of interest at 1 µm interval. The number of DAPI-containing nuclei with or without GR co-localization was manually counted. GR nuclear localization was expressed as number of nuclei showing colocalization relative to the sum of nuclei with the presence or absence of co-localization^24,25^.

### Quantification of brain-derived neurotrophic factor (BDNF)

Methods similar to GR immunohistochemistry were used for BDNF visualization. The primary antibody consisted of mouse anti-BDNF antibody (1:300, Developmental Studies Hybridoma Bank, University of Iowa). The secondary antibody was DyLight-488 horse anti-mouse IgG (1:1,000, Abcam).

35–40 confocal slices were collected per region of interest at 1 µm interval. Three slices (#6, #12, and #18) were chosen for the subsequent analysis. The fluorescent signal derived from BDNF was converted to an 8-bit image such that the intensity of the signal varied between 0 (no signal) to 255 (maximum). The number of pixels at each intensity was then measured. Out of all pixels with non-zero intensities, 99.2 ± 0.2% exhibited intensities lower than 150, demonstrating that the confocal images were not saturated. The normative histogram for each experimental group is depicted in Supplementary Fig. 2. The relationship between intensity and the corresponding cumulative number of pixels was modeled using an exponential plateau function, Y = Ymax (1 − e^(−k×X)^). Ymax in this equation estimates asymptote for the number of pixels with positive staining values. On the other hand, k, or rise constant, estimates relative preponderance of pixels with lower intensity values in the histogram.

### Western blots

The brain from a subset of animals was harvested after rapid decapitation, frozen in liquid nitrogen, and stored in – 80 °C. Harvested brains were sectioned in coronal planes at 100 µm thickness and mounted on glass slides. The basolateral amygdala was collected from the sections using bilateral micro-punches (Bregma − 2.04 to − 3.36 mm). Collected tissue was lyzed in the presence of protease inhibitors, homogenized and centrifuged at 4 °C, and then used to estimate the amount of GR and BDNF amount relative to protein product of an internal housekeeping gene (β-actin). Equivalent amounts of post-lysis samples were loaded on pre-cast sodium dodecyl sulfate polyacrylamide gel and allowed to electrophorese for 45 min at 145 V. Gels were then transferred onto a membrane (Trans-Blot Turbo Cassette; BioRad), cut in accordance to the expected size of the protein products, and blocked in 5% bovine serum albumin. Membrane fragments were incubated with primary antibodies for 14 h, washed, and incubated with secondary antibodies for 1 h. All antibody solutions were diluted with 5% bovine serum albumin. Secondary antibodies were visualized. The relative intensity of the protein bands was quantified using NIH ImageJ (version 1.50b; NIH)^26^.

For GR protein (90–95 kDa), anti-GR rabbit IgG was used as a primary antibody (1:500, Santa Cruz) and goat anti-rabbit IgG as secondary antibody (1:2,000, Santa Cruz). For BDNF (14–15 kDa), anti-BDNF mouse IgG was used as a primary antibody (1:300, University of Iowa) and donkey anti-mouse IgG as secondary antibody (1:2,000, Santa Cruz). For β-actin (37 kDa), anti-β-actin mouse IgG was used as a primary antibody (1:1,000, Santa Cruz) and donkey anti-mouse IgG as secondary antibody (1:2,000, Santa Cruz). The relative intensity of the protein bands was quantified using ImageJ software^26^.

### Quantification of DNA methylation

Basolateral amygdala tissue was isolated using microdissection, as described in the previous section. DNA was isolated using a commercial kit (AllPrep DNA/RNA kit, Qiagen), and shipped to a commercial vendor for methylation quantification (QIAGEN GmbH). We assessed methylation status using direct bisulfite DNA sequencing. Bisulfite-treated samples were amplified through polymerase chain reaction using primers targeted at CpG-rich regions within GR. Amplified products were purified using a gel extraction kit and sequenced using the reverse primer. Peak values of A and G were quantified on the electopherogram. For each CpG site within the target region methylation status was quantified using ratio between G relative to the sum of A and G. Universally methylated and unmetlylated standards were run concurrently. Three CpG sites on GR promotor were quantified, and average methylation across three sites was used as an endpoint. The amplicon and four sites were as follows (CpG sites underlined):

GTTCTGCGGCACGCCCACTTCTAGCAGATAAGGCCGGGCGGGCGA.

### Statistics and analysis

Outliers were identified and removed using the ROUT method with a maximum false discovery rate set at 1%^27^. The experimental design included two treatments, namely, stress exposure and housing conditions. The main effects of these experimental conditions were estimated using a two-way analysis of variance. Omnibus post-hoc comparisons were not conducted in order to avoid multiple pair-wise contrasts^28^.

Experiments were designed to determine if the effects of stress were dependent on housing conditions. In keeping with this a priori interest, two orthogonal planned comparisons were constructed using independent sample t-test^28^. These were (1) between non-stressed controls and stressed animals under animal-facility rearing (AFR versus MS); and (2) between non-stressed controls and stressed animals reared in enriched housing (EE versus MSEE). Information about the number of samples (n) is included in the figures and figure legends.

## Results

### Bodyweight

Both maternal separation and exposure to enriched housing were terminated before weaning of experimental subjects at 3 weeks of the age. We started by analyzing the weight of animals at weaning and gain in weight between weaning and sacrifice at 9–10 weeks of age.

Maternal separation did not cause significant difference in weaning weight in standard animal facility rearing conditions (Supplementary Fig. 1A; Δx̅ = − 2.1 ± 3.0 g; t_14_ = 0.69, *p* = 0.499). These two groups also did not exhibit a significant difference in weight gain during adulthood (Supplementary Fig. 1B; Δx̅ = 14.9 ± 11.6 g; t_14_ = 1.29, *p* = 0.218). In contrast, stressed animals had significantly lower weight at weaning compared to corresponding controls when animals were raised in the enriched housing (Δx̅ = − 7.0 ± 2.1 g; t_14_ = 3.30, *p* = 0.005). Stressed animals in enriched housing also gained lesser weight in adulthood compared to corresponding unstressed controls (Δx̅ = − 22.00 ± 10.06 g; t_14_ = 2.19, *p* = 0.046).

Analysis of variance showed significant main effect of environmental enrichment on body weight at weaning (Δx̅ = − 8.8 ± 1.8 g; *p* < 0.001) and for weight gain during adulthood (Δx̅ = − 23.7 ± 7.7 g; *p* < 0.004). Maternal stress presented with a significant main effect on body weight at weaning (Δx̅ = − 4.5 ± 1.8 g; *p* = 0.019). However, this effect became statistically non-significant for body weight gain during adulthood (Δx̅ = − 3.5 ± 7.7 g; *p* = 0.648).

### Glucocorticoids

Glucocorticoid tone was measured through the wet weight of adrenal glands during sacrifice and baseline corticosterone levels during adulthood (Fig. 1). Adrenal weights were normalized to body weight of the respective animals (mg/kg body weight). Maternal separation did not cause statistically significant differences in normalized adrenal weights, in absence (Δx̅ = 8.21 ± 7.47 mg/kg; t_9_ = 1.1, *p* = 0.300) or presence (Δx̅ = − 3.34 ± 11.7 mg/kg; t_13_ = 0.29, *p* = 0.780) of enriched housing. When reared in standard housing environment, maternal separation significantly increased baseline corticosterone levels (Δx̅ = 23.4 ± 9.6 ng/ml; t_9_ = 2.43, *p* = 0.038). In contrast, effects of stress on basal corticosterone were not statistically significant when animals were reared in enriched housing before weaning (Δx̅ = 25.2 ± 17.9 ng/ml; t_13_ = 1.41, *p* = 0.183).

Analysis of variance revealed statistically insignificant effects of pre-weaning stress and enrichment on normalized adrenal weight during adulthood (*p* = 0.750 for maternal separation and *p* = 0.394 for environmental enrichment). Stress increased the amount of circulating basal corticosterone (Δx̅ = 24.3 ± 11.3 ng/ml; *p* = 0.042), while enrichment did not cause any statistically significant change (Δx̅ = 9.9 ± 11.3 ng/ml; *p* = 0.390).

### Anxiety-like behavior

**Figure 2:**
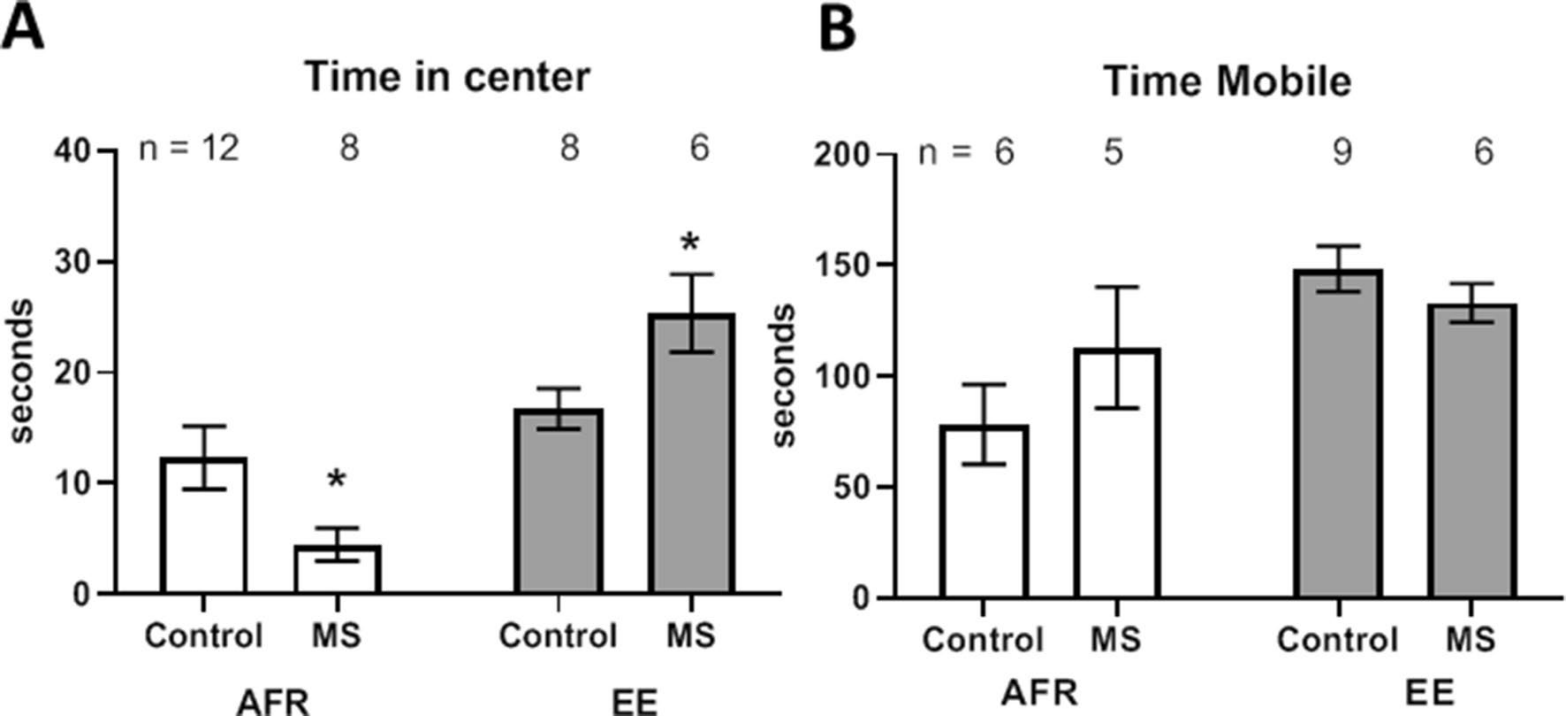
(**A**) Effects of MS and EE on time spent in the anxiogenic center of an open field. *p < 0.05. n refers to number of animals. (**B**) Effects of MS and EE on total locomotion, measured as time sent being mobile in the arena.

The effect of pre-weaning stress and enrichment on adult anxiety-like behavior was measured using the time spent in the anxiogenic center of an open field (Fig. 2A). Stress and enrichment caused contrasting effects on anxiety-like behavior. Maternal separation reduced exploration on anxiogenic center (Δx̅ = − 7.8 ± 3.7 s; t_18_ = 2.1, *p* = 0.050), while environmental enrichment increased such exploration (Δx̅ = 8.583 ± 3.692 s; t_12_ = 2.32, *p* = 0.038).

Analysis of variance showed a significant main effect of enrichment, demonstrating that enrichment increased time spent in center of the open field (Δx̅ = 12.6 ± 2.7 s; *p* < 0.001. Main effect of stress did not reach statistical significance (Δx̅ = 0.4 ± 2.7 s; *p* = 0.891).

Maternal separation did not significantly affect locomotion, as measured through time spent being mobile in the arena, both in absence (Fig. 2B, Δx̅ = 3.7 ± 29.0 s; t_9_ = 0.1, *p* = 0.902) and presence of environment enrichment (Δx̅ = − 15.3 ± 14.5 s; t_13_ = 1.1, *p* = 0.400).

### Spine density of principal basolateral amygdala neurons

**Figure 3:**
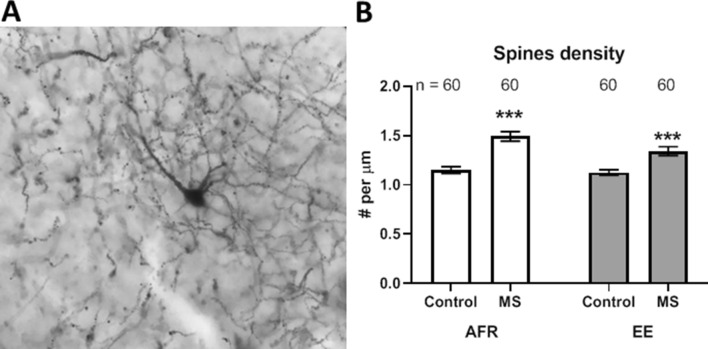
(**A**) A representative image depicting a Golgi stained neuron at 400× optical magnification. (**B**) Effects of MS and EE on the spine density of principal neurons in basolateral amygdala. ***p < 0.001. n refers to number of neurons.

The density of dendritic spines on principal basolateral amygdala neurons was measured in Golgi-stained preparations (Fig. 3A). Spine density was quantified in adulthood as a function of pre-weaning stress and enrichment (Fig. 3B). Maternal separation caused a significant increase in spine density, both in absence of enrichment (Δx̅ = 0.34 ± 0.06/µm; t_118_ = 5.81, *p* < 0.001) and in presence of enrichment (Δx̅ = 0.22 ± 0.05/µm; t_118_ = 4.07, *p* < 0.001).

Analysis of variance revealed contrasting main effects of stress and enrichment on the spine density. While maternal separation caused an increase in spine density (Δx̅ = 0.28 ± 0.04/µm; *p* < 0.001), environmental enrichment reduced it (Δx̅ = − 0.09 ± 0.04/µm; *p* = 0.027).

### Intra-nuclear glucocorticoid receptors (GR)

**Figure 4:**
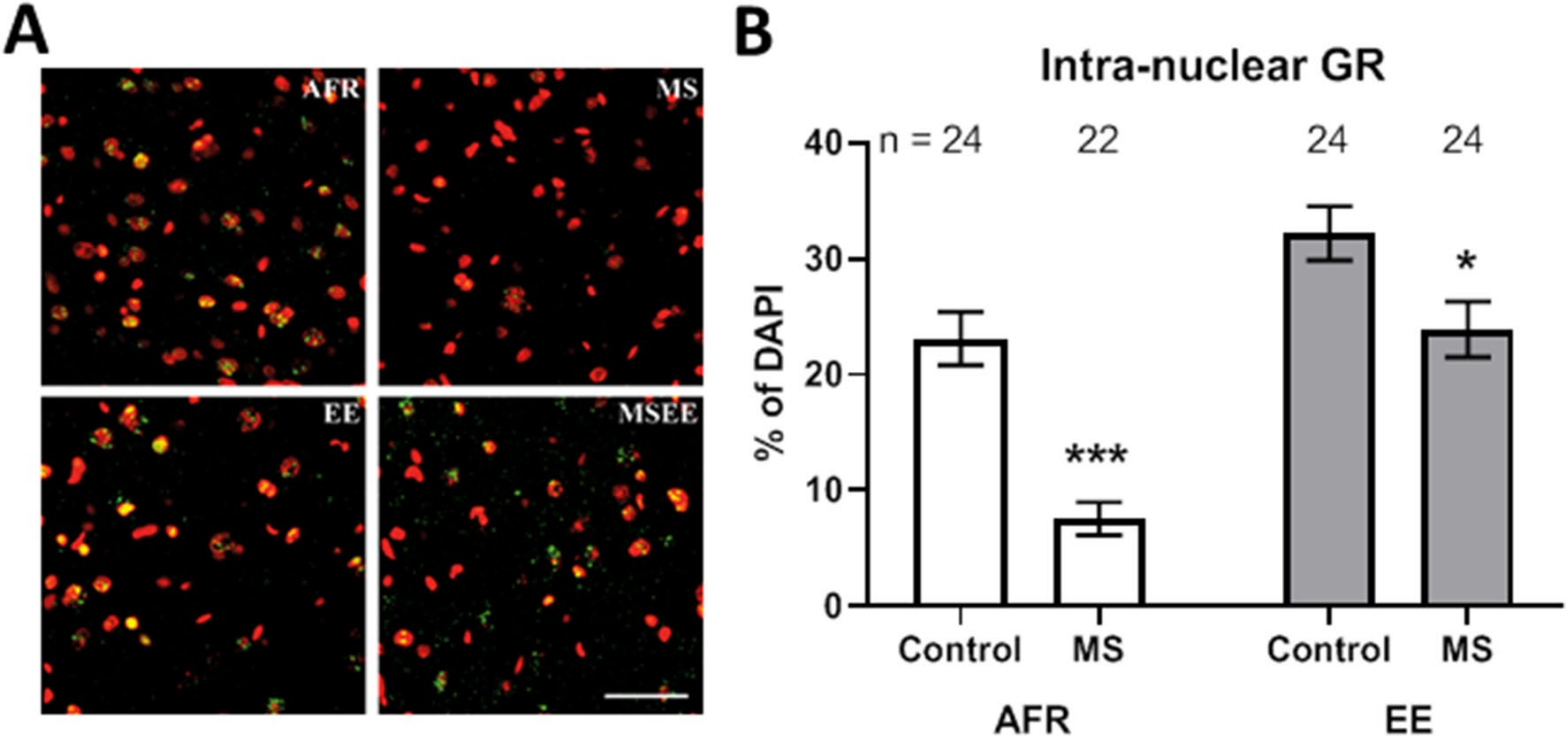
(**A**) Representative images depicting labelling for glucocorticoid receptors (green) and DAPI (psudocolored red). Scale bar = 50 µm. (**B**) Effects of MS and EE on the percentage of basolateral amygdala cells exhibiting intra-nuclear localization of glucocorticoid receptors (GR). *p < 0.05; ***p < 0.001. n refers to number of sections.

The extent of intra-nuclear localization of GR was quantified as percentage of DAPI-positive total nuclei (Fig. 4A,B). Maternal separation before weaning resulted in statistically significant reduction in intra-nuclear GR during adulthood, both in absence (t_44_ = 5.62, *p* < 0.001) and presence (t_46_ = 2.46, *p* = 0.018) of pre-weaning environmental enrichment. Magnitude of the stress effect was more pronounced in absence of enrichment (Δx̅ = − 15.5 ± 2.8%) compared to that in presence of enrichment (Δx̅ = − 8.3 ± 3.4%).

Analysis of variance showed opposing main effects of stress and enrichment on intra-nuclear GR localization. Maternal separation reduced movement of GR within nuclei (Δx̅ = 11.9 ± 2.2%; *p* < 0.001). In contrast, environmental enrichment increased intra-nuclear localization (Δx̅ = − 12.7 ± 2.2%; *p* < 0.001).

**Figure 5:**
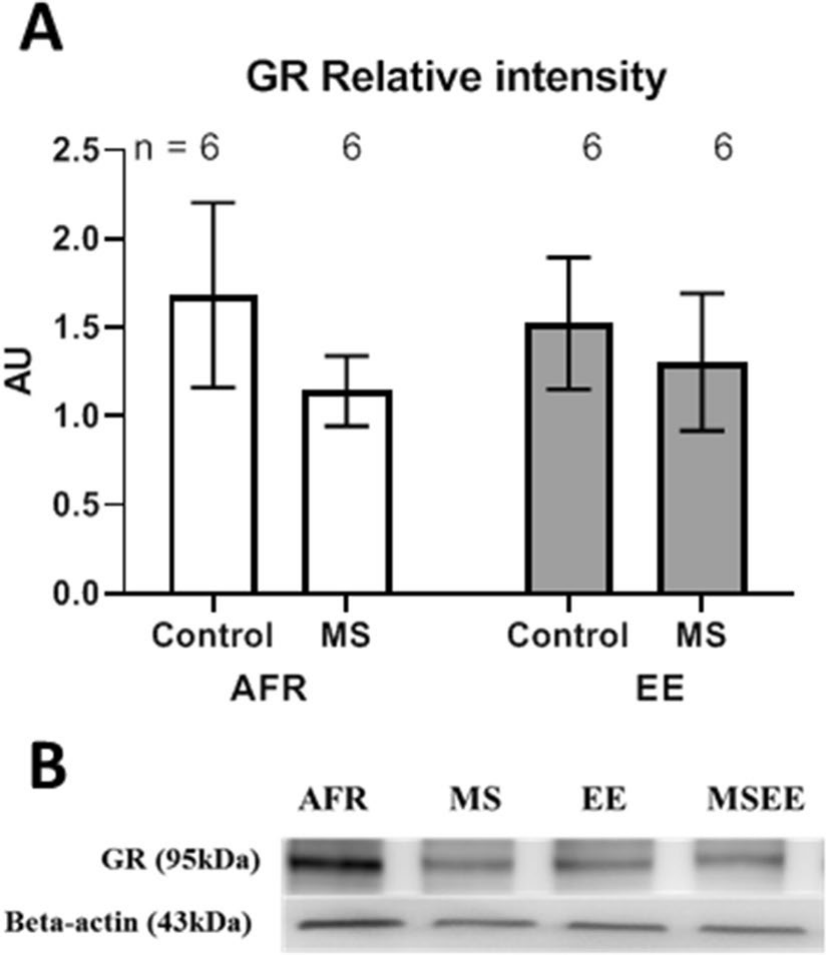
(**A**) Effects of maternal separation (MS) and environmental enrichment (EE) on amount of total GR protein. (**B**) Representative Western blot images for GR and an internal control (Beta-actin). n refers to number of animals.

**Figure 6:**
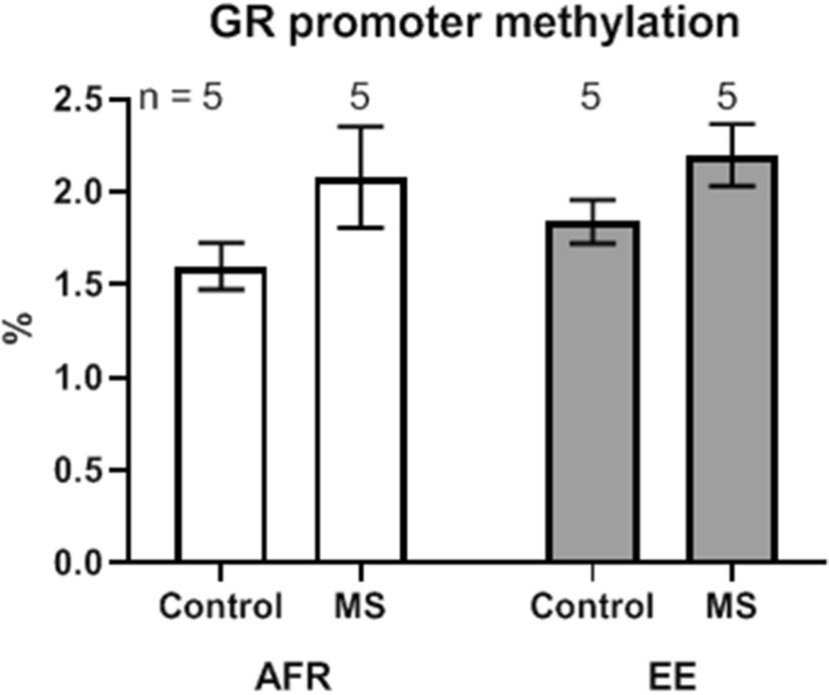
Effects of maternal separation (MS) and environmental enrichment (EE) on percentage DNA methylation at the GR promoter site. n refers to number of animals.

Western blot analysis of total GR content within basolateral amygdala tissue did not reveal significant differences between stressed and non-stressed groups (Fig. 5; t_10_ = 0.97, *p* = 0.355 in the absence of enrichment and t_10_ = 0.40, *p* = 0.670 in the presence of enrichment). Similarly, main effects of stress and enrichment did not exhibit statistical significance (*p* = 0.339 and 0.995, respectively). Genomic DNA from basolateral amygdala of a subset of animals was also probed for DNA methylation status on five sites on the GR promoter (Fig. 6). This analysis showed very weak DNA methylation of GR promoter in the basolateral amygdala across all groups (x̅ = 1.9 ± 0.1%; range from 1.2 to 2.8%). Planned comparisons did not reveal significant effects of stress on absence or presence of enrichment (t_8_ = 1.60, *p* = 0.149 and t_8_ = 1.76, 0.116, respectively; Δx̅ < 0.5%).

### Brain-derived neurotrophic factor (BDNF)

**Figure 7:**
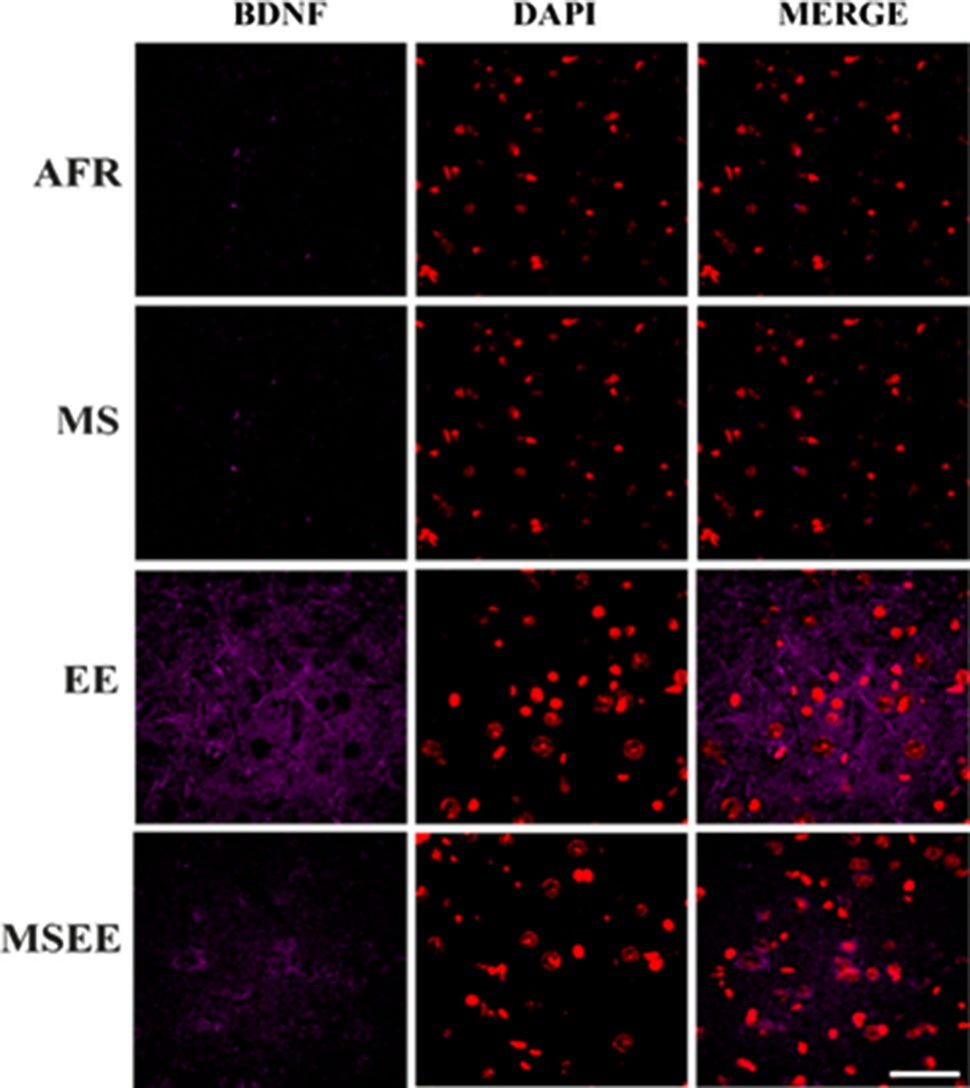
Representative images depicting labelling for BDNF (psudocolored magenta) and DAPI (psudocolored red). Scale bar = 50 µm.

The amount of BDNF across experimental groups was quantified as a function of staining intensity (Fig. 7). The percentage number of pixels at each fluorescent intensity from 1 to 255 was quantified. These values were fitted with an exponential plateau function to calculate the rise constant and Ymax for each image. Inter-group variation in these endpoints was then analyzed.

**Figure 8:**
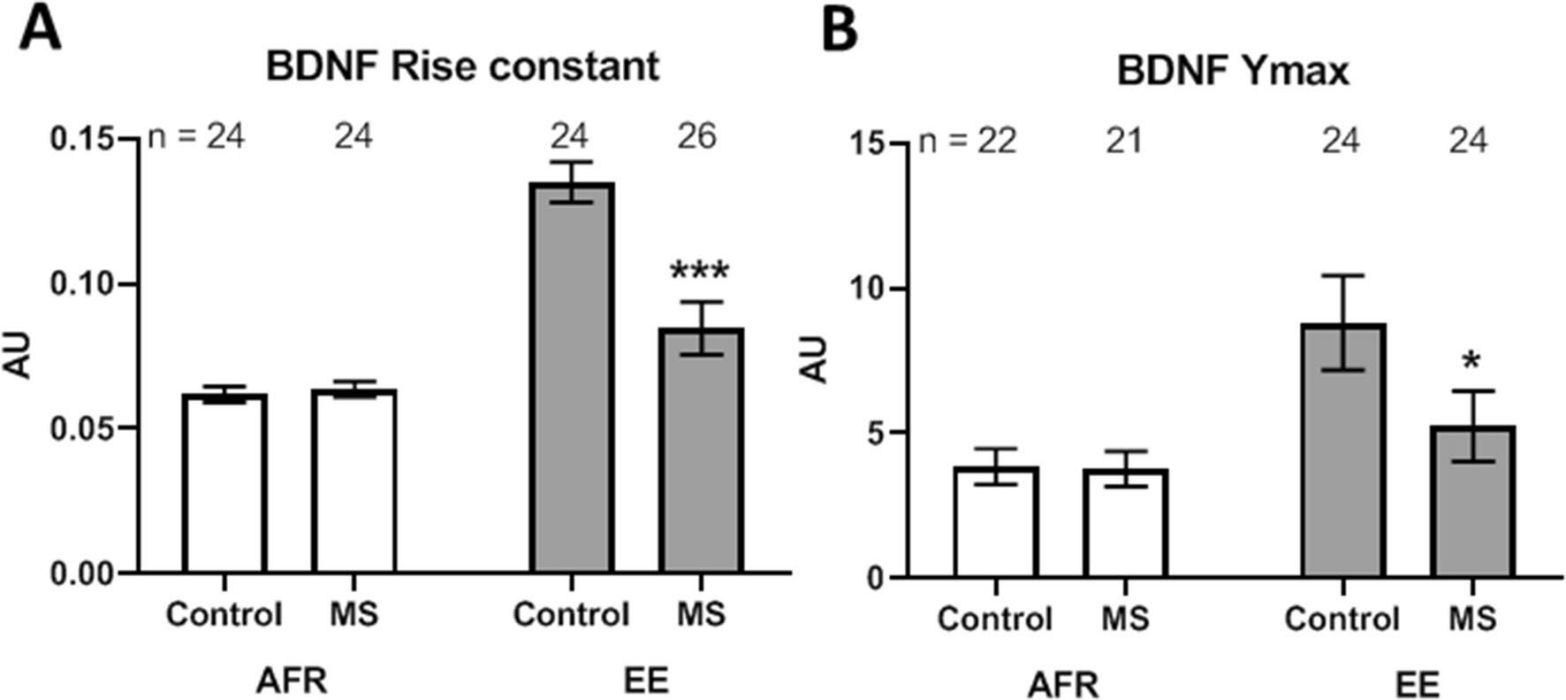
(**A**) Effects of MS and EE on rise constant of brain-derived neurotrophic factor signal within the basolateral amygdala and its asymptote (**B**, Ymax). *p < 0.05; ***p < 0.001. n refers to number of sections.

Preponderance of signal with lower intensity within the total amount of fluorescence was estimated using rise constant of the exponential plateau function. Pre-weaning maternal separation did not cause significant change in rise constant of BDNF (Fig. 8A; Δx̅ = 0.002 ± 0.004; t_46_ = 0.49, *p* = 0.623). Rise constant of BDNF in non-stressed controls was significantly higher than stressed groups when animals were provided with pre-weaning enrichment (Δx̅ = − 0.050 ± 0.012; t_48_ = 4.31, *p* < 0.001). Main effects during analysis of variance showed that environmental enrichment increased rise constant (Δx̅ = 0.47 ± 0.006; *p* < 0.001). In contrast, maternal separation decreased rise constant (Δx̅ = 0.024 ± 0.006; *p* < 0.001).

Total extent of the fluorescence staining was estimated using Ymax, or the asymptote, of the exponential plateau function. Maternal separation did not cause a significant change in Ymax of BDNF in absence (Fig. 8B; Δx̅ = − 0.076 ± 0.867; t_41_ = 0.09, *p* = 0.931) of environmental enrichment. Ymax in non-stressed controls was significantly higher than stressed groups when animals were provided with pre-weaning enrichment (Δx̅ = − 4.491 ± 1.813; t_46_ = 2.48, *p* = 0.017). Analysis of variance revealed significant increase Ymax due to environmental enrichment (Δx̅ = 3.211 ± 1.151; *p* = 0.006). Main effect of maternal separation was not statistically significant (Δx̅ = − 1.818 ± 1.151; *p* = 0.188).

**Figure 9:**
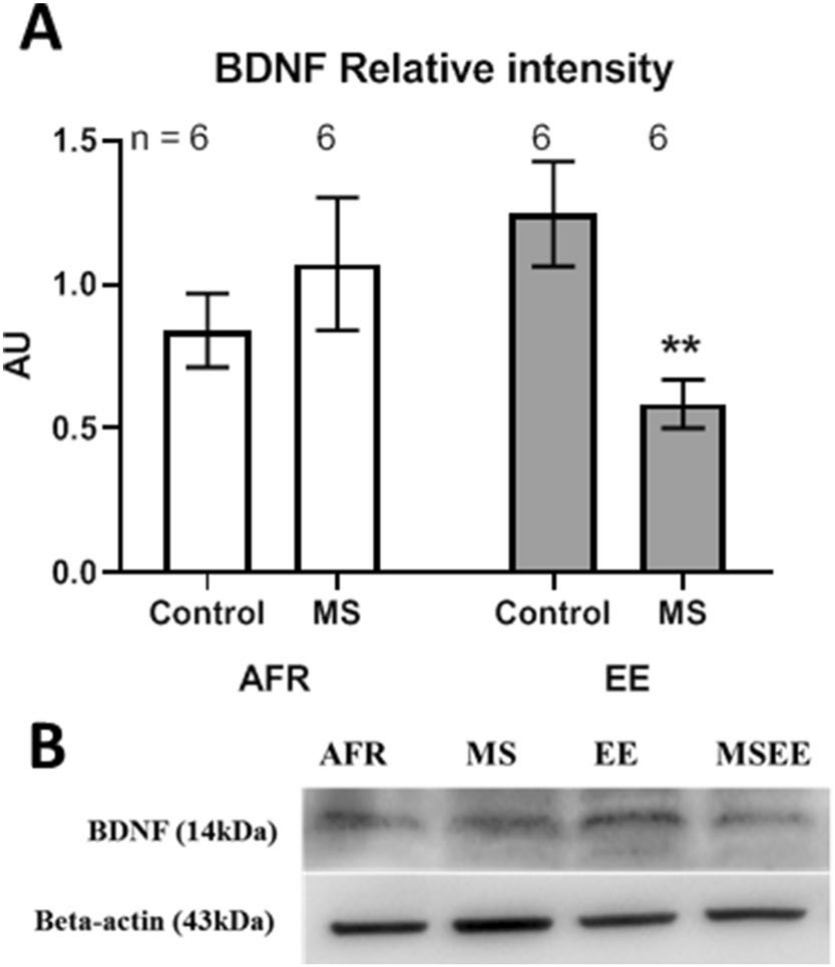
(**A**) Effects of maternal separation (MS) and environmental enrichment (EE) on amount of total BDNF protein. (**B**) Representative Western blot images for BDNF and an internal control (Beta-actin). n refers to number of animals.

Congruent with Ymax, relative intensity of BDNF protein during Western blot analysis showed lack of significant difference due to maternal stress in absence of enrichment (Fig. 9; Δx̅ = 0.23 ± 0.26; t_10_ = 0.87, *p* = 0.404) and a significant decrease in the stressed group in presence of enrichment (Δx̅ = − 0.66 ± 0.20; t_10_ = 3.28, *p* = 0.008). Analysis of variance for relative amount of BDNF did not have significant main effects of either enrichment (Δx̅ = − 0.041 ± 0.166; *p* = 0.809) or stress (Δx̅ = − 0.215 ± 0.166; *p* = 0.210).

## Discussion

Exposure to early life stress causes potentiation of the stress response and related behaviors in adulthood^29–34^. In congruence with these earlier observations, results in this report show an increase in anxiety-like behavior and basal tone of hypothalamus–pituitary–adrenal (HPA) axis. Adult animals that were exposed to maternal separation before weaning exhibited more glucocorticoids circulating in the bloodstream at baseline and reduced spatial exploration of an anxiogenic environment. These detrimental effects occurred in parallel to an increase in the density of dendritic spines in the basolateral amygdala (BLA), a brain region known to be involved in stress-induced anxiogenesis^35^. These changes reflect a long-lasting footprint of early life stress through sustained neuroendocrine and neuroplastic reconfiguration into adulthood.

Interestingly, the effects of early life stress are dependent upon the housing environment of the mother–pup dyad during stress exposure^36–38^. Maternal separation stress during early life caused an increase in anxiety-like behavior in adulthood, yet such stress-induced anxiogenesis was prevented by simultaneous exposure to an enriched environment. While maternal separation still caused an increase in the dendritic spine density of BLA neurons, the magnitude of this effect was much reduced from a Cohen's d of 1.1 in the non-enriched cohort to 0.7 in the enriched cohort. The general theme of opposing effects of stress and enrichment was also reflected in contrasting the main effects of these treatments during the analysis of variance. The maternal separation had the main effect of more considerable anxiety-like behavior and denser BLA dendritic spines. In contrast, environmental enrichment had the main effect of reduced anxiety-like behavior and lowered spine density of the BLA neurons. These results suggest that the relationship between early-life stress and adult stress response as well as anxiety-like behavior is dependent on the immediate environment of early life. Individuals exposed to stress in early life take vastly different neuroendocrine trajectories, neuroplasticity and receptor regulation depending on the environmental context of the early housing of mother and offspring.

The modifying role of environment on the effects of stress is a recurring theme in a large body of scientific work^14,39–41^. For example, animals exposed to chronic stress in adulthood develop anxiety if they live in standard laboratory housing but not in environmentally enriched housing^12^. Similarly, enhanced anxiety-like behavior in adulthood brought about by early maternal separation can be effectively rescued by peripubertal environmental enrichment or by a relatively short period of enrichment later in adult life^13^. The effects of naturally occurring variations in maternal care also demonstrate such a contingency of effects on adult behavior and stress response^38,42,43^. Pups raised by low maternal care mothers show increased anxiety-like behavior, and hyper-responsive HPA system^32,44^, and this effect can be effectively reversed by providing environmental enrichment to the offspring after weaning^14^. In contrast to these earlier studies, stress and enrichment were concomitant during early life in the current study. Maternal separation occurred whilst the mother and the pups were living in either improvised laboratory housing or in enriched housing. This is an important distinction because dyadic interactions between the mother and the child play a crucial role in the development of behavior in many species^45–47^. In rats, maternal presence modulates stress response and shapes  fear behavior in the pups^5,38^. In humans, lower quality of maternal care results in increased anxiety and disturbed social interactions during adulthood, leading to reduced lifespan and increased disease burden through allostatic load^48–50^. Results presented here suggest that modification of the environment where maternal care occurs can successfully preclude the harmful effects of the loss of maternal care.

Maternal separation caused a significant reduction in the number of BLA neurons manifesting intra-nuclear localization of glucocorticoid receptors (GR). This stress-induced suppression of GR nuclear localization, became blunted when animals were raised in the enriched housing. The mean difference between unstressed and stressed animals halved in the presence of enrichment, with a corresponding reduction in standardized effect size measured through Cohen’s *d* from 1.7 to 0.7. Glucocorticoids secreted by adrenal glands can readily cross blood–brain barrier and plasma membranes of neurons. These steroid molecules then act as ligands to cytosolic GR, initiating their traversal to the inside of the nucleus where GR acts as transcription factor by binding to GRE and eventually change the molecular milieu of the neurons^51,52^. Our results suggest that maternal separation reduces the ability of glucocorticoids to initiate downstream molecular changes in the BLA neurons despite the greater concentration of the circulating ligand. Simultaneous exposure to environmental enrichment partially reversed this phenomenon. The absence of statistically significant or numerically robust differences in GR total protein suggests that inter-group differences were not driven by expression of the GR but were mainly affected by nuclear localization of the GRs. Congruently, neonatal isolation for a week or brief maternal separation spanning two weeks does not alter GR expression within BLA^53^, in contrast to the known effects of early life stressors on hippocampal GR expression. Stressors applied during adulthood are known to decrease total GR expression within BLA^54^. In contrast, GR expression increases after chronic unpredictable stress and treatment with glucocorticoid analogs. Thus, it is likely that stress exposure during developmentally labile early life causes persistent rerouting of the biochemical steps involved between binding of GR to its ligand and its eventual transfer inside the nucleus to initiate transcriptional regulation. Apart from binding to glucocorticoids, GRs can also influence the secretion of their own ligands from the adrenals^55^. A genetic knockout of forebrain glucocorticoid receptors, for example, increases activity of hypothalamus–pituitary–adrenal axis^56^. Thus, it is plausible that reduced nuclear localization of GRs after maternal separation is responsible for hypercortisolism in the stressed group. Moreover, successful recruitment of GRs within BLA is known to be required from memory consolidation and facilitation of spatial memory during stressful events^57^. Thus, our results suggest that prior exposure to maternal separation might compromise the substrates that are later used for the mnemonic functions.

The effects of maternal separation and environmental enrichment on the brain-derived neurotrophic factor (BDNF) within BLA have not yet been studied. In other brain regions, BDNF is generally conceived to have a permissive effect of spinogenesis and structural plasticity of the neurons^58–62^. Previous work has established that stressors, including maternal separation, cause a long-lasting increase in dendritic material within BLA^35,63^. This is congruent with current observation that maternal separation increases spine density of BLA neurons. We have earlier shown that environmental enrichment during adulthood prevents dendritic growth in BLA brought about maternal separation^13^. These observations, together with neurotrophic effects of BDNF signaling, would suggest suppression of BDNF within BLA during environmental enrichment. In contrast, pre-weaning environmental enrichment increased the amount of BDNF within adult BLA, as inferred by a greater amount of the integrated signal. Previous studies have shown that binding of BDNF to its TrkB receptors is required for the effects of GRs on memory performance^64^. Thus, BDNF-TrkB signaling and its downstream effects on the extracellular signal-regulated kinase pathway might be a critical conduit for the effects of occupied GRs on molecular plasticity. Consistent with this, we observe that environmental enrichment leads to a diffused BDNF distribution within the BLA as would be expected by cleavage of BDNF to its active form ready to interact with its cognate receptor. This is indicated by larger rise constant for BDNF intensity in the enriched group. Thus, the developmental window at which animals experience enrichment might have important implications for long-term biochemical alterations in BDNF and its partner proteins. The period of maternal separation used in this study encompasses the critical temporal window for the development of the amygdala and its responsiveness to glucocorticoids.

Coincident presence of BDNF and glucocorticoids is required for efficient nuclear translocation of the GRs^20,65,66^. The presence of BDNF alone does not initiate GR nuclear localization. Yet, BDNF enhances the potency of GR mediated genomic response by increasing its nuclear transport when bound with glucocorticoids. It is then likely that a high BDNF environment within BLA, provided by enriched environment creates an enabling molecular backdrop for efficient GR signaling. In the non-enriched animals, lack of robust BDNF diminishes the ability of stress-induced glucocorticoids to drive genomic response and ensuing plasticity through GR, as seen in feeble nuclear localization of GRs in maternally separated cohort.

Rat pups are born altricial, dependent on maternal care for protection and nourishment. Moreover, the developing brain of pups is highly responsive to variations in the maternal environment. For example, the presence of a mother can obliterate stress endocrine activation during physically painful stimuli and alter the course of subsequent conditioned responses^5^. In this study, environment enrichment was provided all through the pre-weaning stage, including early postnatal periods where eyes of the pups did not open and when the mother remained the main conduit for environmental effects on the pups. It is possible that enrichment induces changes in maternal interaction with her pups during the pre-weaning period. For example, pre-reproductive enrichment of females is known to increase pup-oriented behaviors to later offspring^67^. Hence, the effects reported in this manuscript could have risen from both direct sensory enrichment of pups as well as indirect enrichment effects of dams that secondarily reached the pups through maternal care. Our data does not delineate the relative influence of these sources. Nonetheless, the present data demonstrate the holistic value of early life intervention on long-lasting neural and behavioral changes.

## Conclusions

Our paper shows for the first time that early environment can play a decisive role in projecting stress-vulnerability and stress-resilience in individuals during adult life. This contrasting outcome is driven at multiple levels, from behavior, anxiogenesis to physiological stress-response through amygdala neuroplasticity. Additionally, we show that the underlying cellular context of stress resilience and vulnerability, driven by early housing environment, involves corticosterone-receptor localization within the nucleus as well as cellular regulation of BDNF, a neurotrophic factor known to influence neuronal spine-plasticity.

## Supplementary information

Supplementary Information.

Supplementary Figure 1.

Supplementary Figure 2.
