# Author Correction: Early‑life short‑term environmental enrichment counteracts the effects of stress on anxiety‑like behavior, brain‑derived neurotrophic factor and nuclear translocation of glucocorticoid receptors in the basolateral amygdala

**DOI:** 10.1038/s41598-020-74322-3

**Published:** 2020-10-06

**Authors:** Akshaya Hegde, Shruti Suresh, Rupshi Mitra

**Affiliations:** School of Biological Sciences, Nanyang Technological University, Singapore, 637551 Singapore

Correction to: *Scientific Reports* https://doi.org/10.1038/s41598-020-70875-5, published online 20 August 2020.

This Article contains an error in Figure 7 where the representative images for MS group are duplicates of the representative AFR images and MSEE DAPI does not match the Merge panel. The correct Figure 7 appears below as Figure 1.

**Figure 1:**
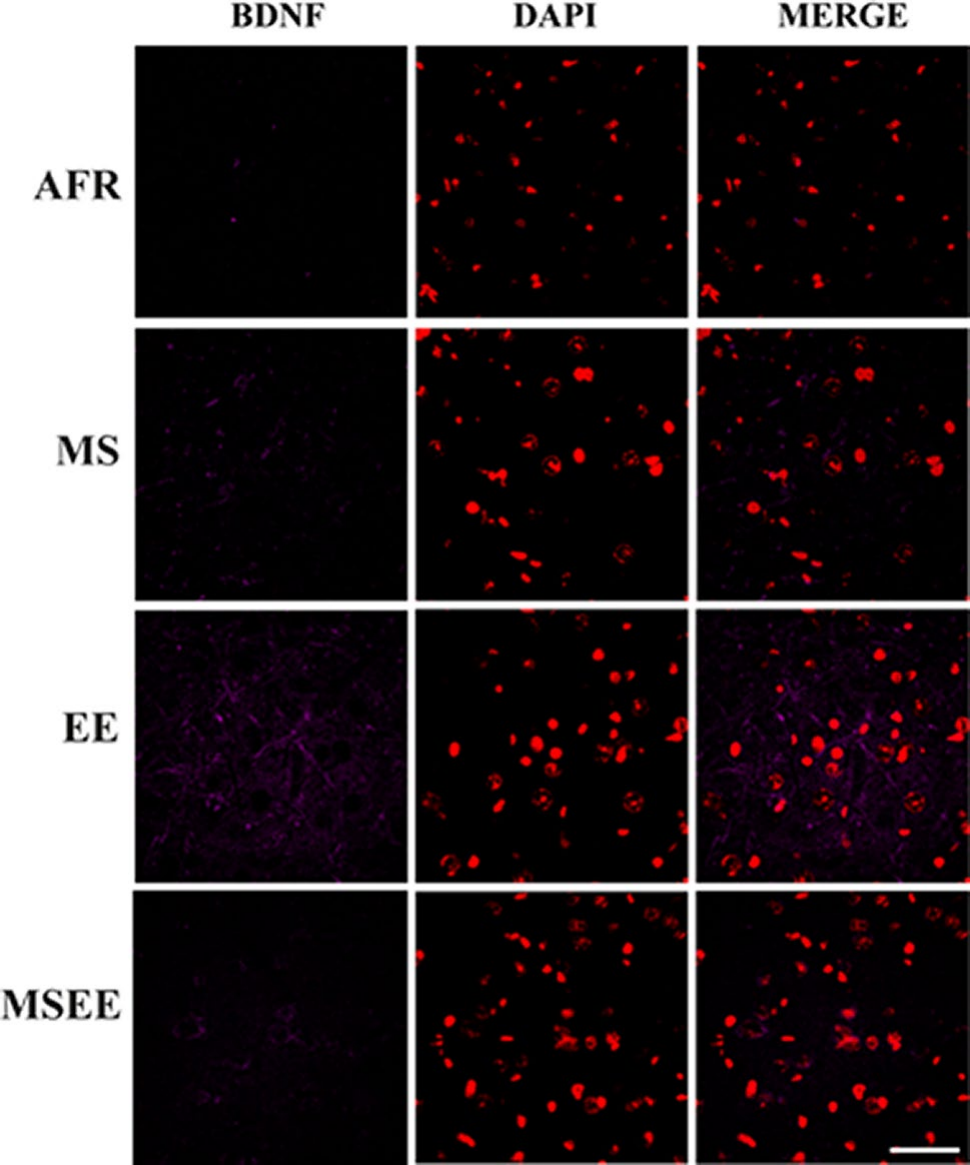
Representative images depicting labelling for BDNF (psudocolored magenta) and DAPI (psudocolored red). Scale bar = 50 µm.

