# Supplementary material for: Early-life short-term environmental enrichment counteracts the effects of stress on anxiety-like behavior, brain-derived neurotrophic factor and nuclear translocation of glucocorticoid receptors in the basolateral amygdala
Source: Sci Rep. 2020 Aug 20;10:14053. doi: 10.1038/s41598-020-70875-5 (PMC7441150; doi:10.1038/s41598-020-70875-5)
Supplement: Supplementary file 1 — Supplementary Information. [file 41598_2020_70875_MOESM1_ESM.pdf]

This is the image of Western blots for Figure 5B.

Early-life short-term environmental enrichment counteracts the effects of stress on anxiety-like behavior, brain-derived neurotrophic factor and nuclear translocation of glucocorticoid receptors in the basolateral amygdala.

Akshaya Hegde, Shruti Suresh, and Rupshi Mitra.

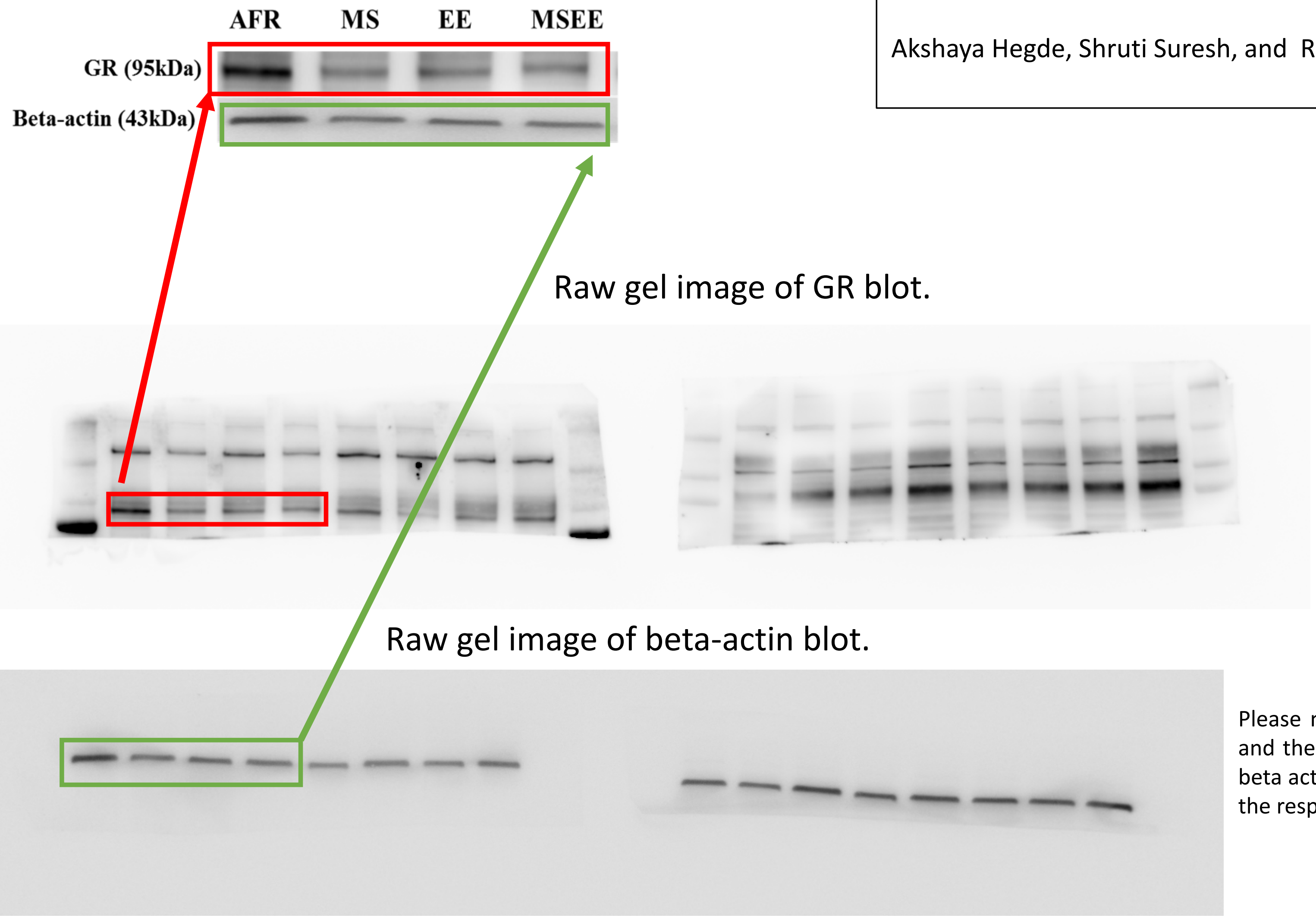

Please note that the blot was probed for GR on day 1 and the same blot was stripped and probed again with beta actin on the next day. Hence, there are 2 blots with the respective images. GR = glucocorticoid receptor.

This is the image of Western blots for Figure 9B.

Early-life short-term environmental enrichment counteracts the effects of stress on anxiety-like behavior, brain-derived neurotrophic factor and nuclear translocation of glucocorticoid receptors in the basolateral amygdala.

Akshaya Hegde, Shruti Suresh, and Rupshi Mitra.

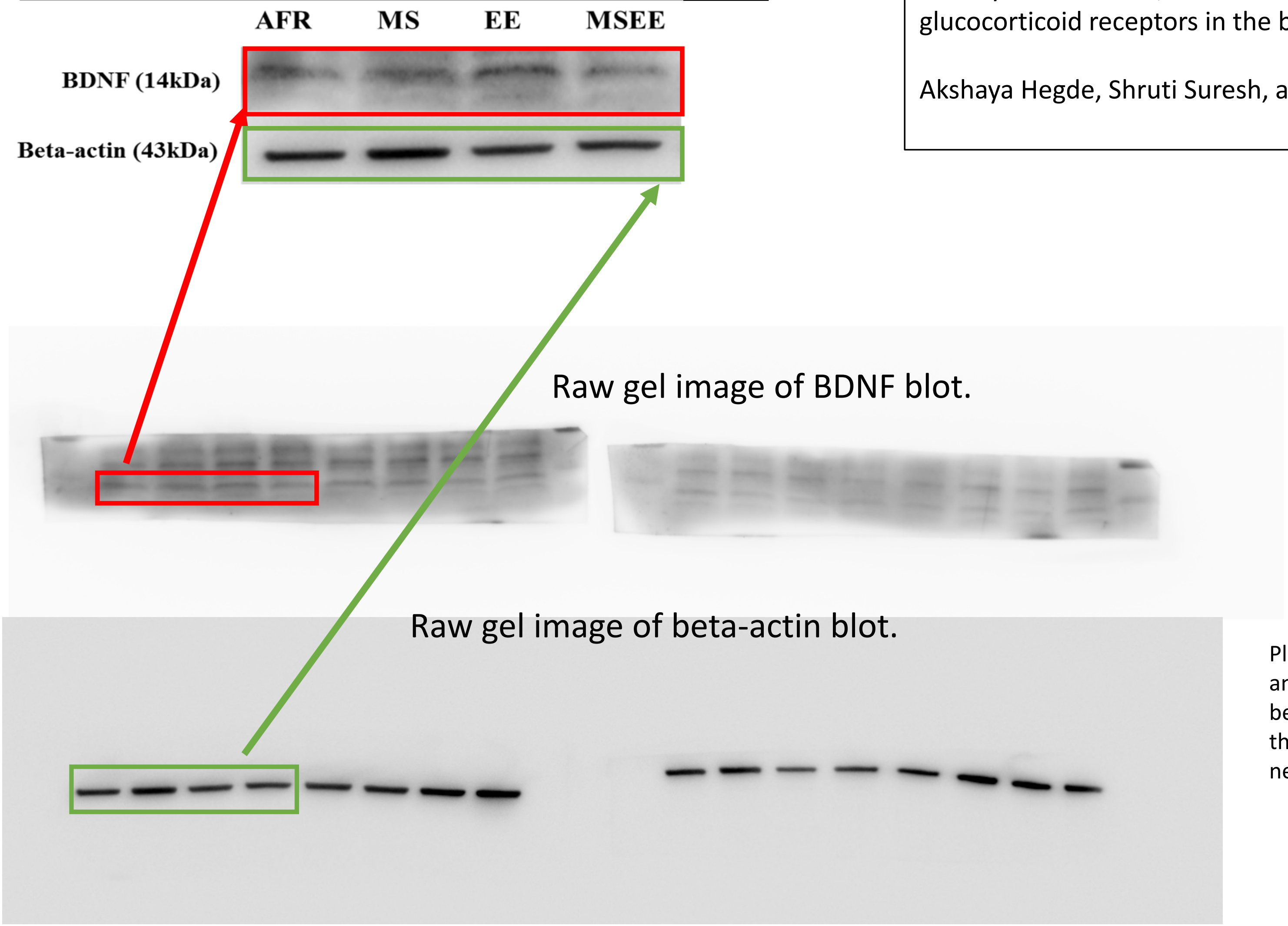

Please note that the blot was probed for BDNF on day 1 and the same blot was stripped and probed again with beta actin on the next day. Hence, there are 2 blots with the respective images. BDNF = Brain-derived neurotrophic factor.
