# Supplementary figures and images for: Early-life short-term environmental enrichment counteracts the effects of stress on anxiety-like behavior, brain-derived neurotrophic factor and nuclear translocation of glucocorticoid receptors in the basolateral amygdala
Source: Sci Rep. 2020 Aug 20;10:14053. doi: 10.1038/s41598-020-70875-5 (PMC7441150; doi:10.1038/s41598-020-70875-5)

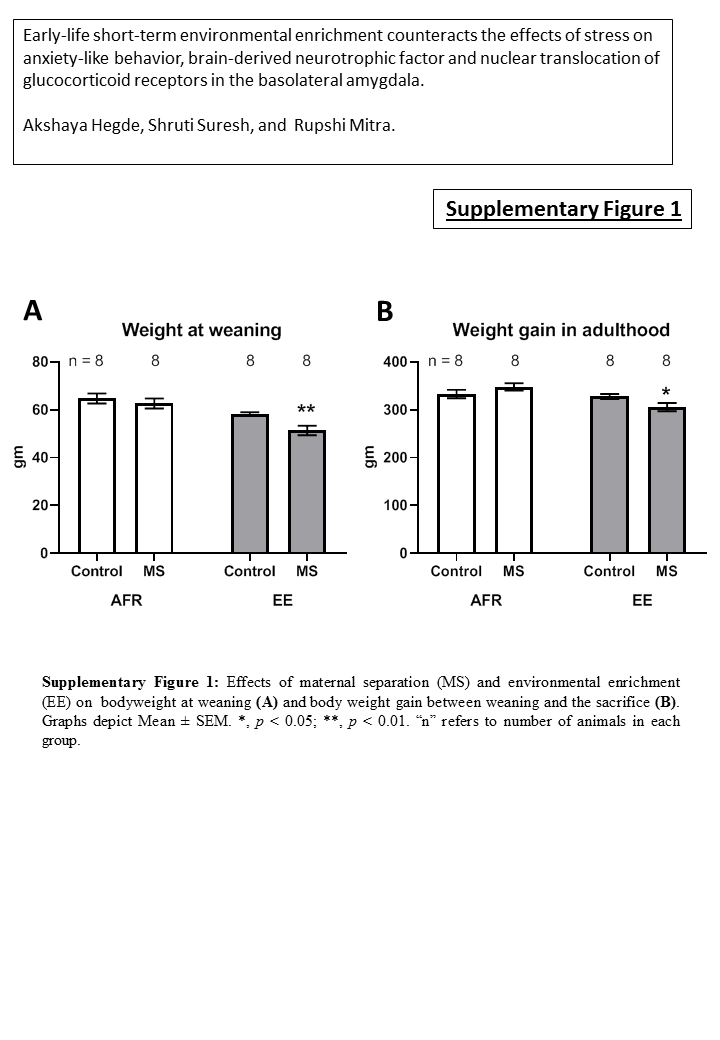

Supplement: Supplementary file 2 — Supplementary Figure 1. [file 41598_2020_70875_MOESM2_ESM.tif]

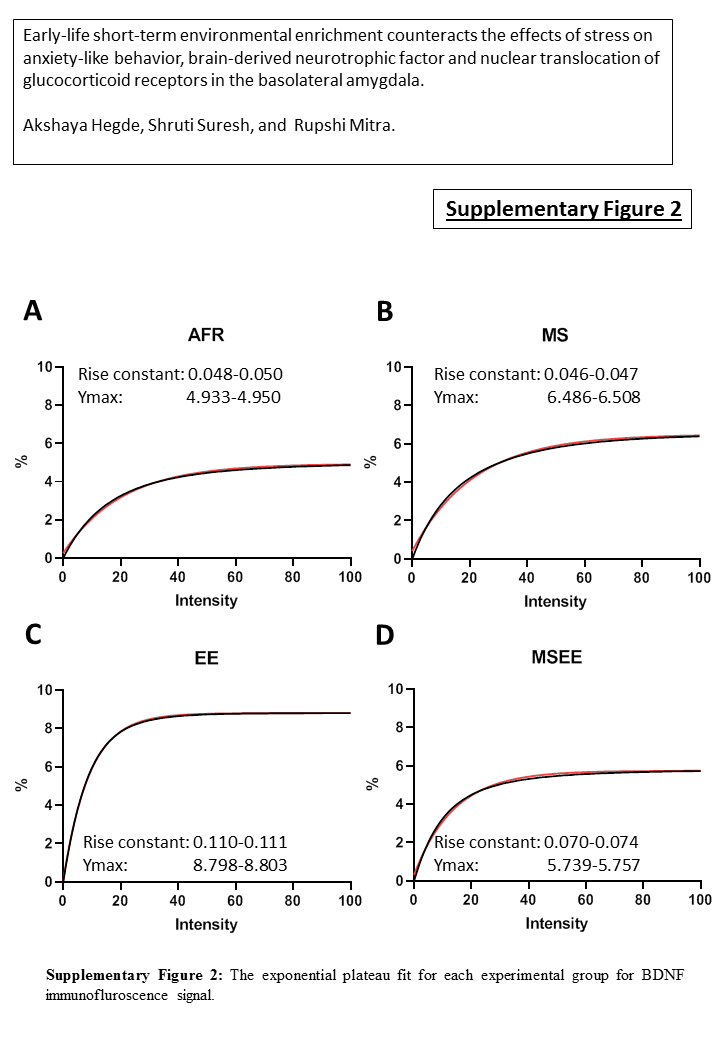

Supplement: Supplementary file 3 — Supplementary Figure 2. [file 41598_2020_70875_MOESM3_ESM.tif]
